# Spatial analysis of under-five children mortality and associated determinants in Ghana: Mapping regional disparities for targeted interventions

**DOI:** 10.1371/journal.pgph.0006332

**Published:** 2026-04-17

**Authors:** Abdul-Karim Iddrisu, Richard Socrate Adzesi, Abubakar Iddrisu Siddiq

**Affiliations:** 1 Department of Statistics, Demography and Population Studies, Faculty of Natural and Applied Sciences, University of Botswana, Gaborone, Botswana; 2 Department of Statistics and Actuarial Science, School of Physical and Mathematical Sciences, University of Ghana, Accra, Ghana; 3 Department of Public Health, Faculty of Medical Science, Presbyterian University, Asante Akyem Agogo, Ghana; The University of Newcastle Australia: University of Newcastle, AUSTRALIA

## Abstract

Child mortality is a critical measure of healthcare performance, with Sustainable Development Goal 3.2 targeting reductions in neonatal and under-five mortality to 12 and 25 deaths per 1,000 live births, respectively, by 2030. However, Ghana’s child mortality rate remains high at 32 deaths per 1,000 live births, with significant regional disparities driven by healthcare access and economic inequalities. This research develops risk maps to identify high-risk regions and determinants, providing valuable insights for targeted interventions to reduce child mortality. We analyzed under-five mortality (U5MR) in Ghana (2022) using data from the 2022 Ghana Demographic and Health Survey. Crude death rates (per 100 live births) were calculated and smoothed with Global Empirical Bayesian (GEB) and Local Empirical Bayesian (LEB) methods. Spatial dependence and clustering were evaluated using Global Moran’s I, Local Indicators of Spatial Association (LISA), and Getis–Ord statistics, and SaTScan statistic. Spatial associations between U5MR and its determinants were assessed using bivariate LISA. Predictor importance was quantified using Gradient Boosting, a nonparametric ensemble learning approach, with interpretability enhanced by SHapley Additive exPlanations (SHAP), including feature importance rankings, partial dependence, and waterfall plots. The Bayesian spatial model, Besag-York-Mollié (BYM2) was also used to further study spatial pattern of child mortality risk in Ghana. In 2022, Ghana’s national under-five mortality rate (U5MR) was 6.4 per 100 live births, with marked regional disparities. Oti, Northern, and Savannah recorded the highest risks, while Greater Accra and Ahafo had the lowest. Spatial analyses (GEB, LEB, SaTScan, LISA, Getis–Ord Gi*) consistently identified high-risk clusters in the northern belt, with a 27% elevated risk in a cluster spanning Oti, Northern, Bono East, and Volta. ANC coverage was the strongest predictor, explaining over 75% of U5MR variation, while caesarean rates also showed strong spatial associations. Persistently high U5MR in high-coverage regions points to gaps in quality of care and underlying structural inequities. Economic and governance factors were less influential, highlighting the critical need for targeted maternal and child health interventions in high-burden areas. The persistent regional disparities in under-five mortality in Ghana, particularly the elevated risks in the northern belt, highlight urgent equity challenges in maternal and child health. Despite national progress, high-risk regions remain underserved in terms of both access to and quality of health services. Antenatal care coverage emerged as the most influential determinant of mortality risk, reinforcing the need to prioritize maternal service delivery in disadvantaged areas. Policy efforts should move beyond national averages to implement region-specific strategies strengthening primary healthcare systems, addressing quality-of-care gaps, and tackling structural barriers such as rural inaccessibility. A more targeted and spatially-informed approach is essential to achieving equitable child survival outcomes across Ghana.

## 1. Introduction

Infant mortality remains a major global public health concern and serves as a key indicator of both healthcare quality and socioeconomic development [1]. Despite notable medical advancements, substantial disparities persist, particularly between high- and low-income countries [2]. In 2020 alone, more than 5 million children under the age of five died, including 2.4 million newborns, with the majority of these deaths deemed preventable [2]. The neonatal period, which accounts for nearly half of all under-five deaths, continues to be the most critical window for child survival. While global neonatal and infant mortality rates have significantly declined since 1990, an estimated 2.3 million newborns and 13,400 children under-five still died each day in 2022, underscoring the urgent need for targeted interventions to reduce preventable child deaths [2]. These persistent inequities in healthcare access and quality emphasize the importance of strengthening maternal and child health systems, particularly in resource-constrained settings.

Sub-Saharan Africa bears the highest burden of infant mortality globally, accounting for more than half of all infant deaths [3]. The region’s average infant mortality rate is approximately 47 deaths per 1,000 live births substantially higher than the rate of 5 per 1,000 observed in high-income countries [1]. Of the ten countries with the highest infant mortality rates worldwide, nine are located in Africa, with Somalia reporting the highest rate at 83.6 deaths per 1,000 live births, followed by the Central African Republic, Equatorial Guinea, and Sierra Leone [4]. This stark disparity is driven by a complex interplay of factors, including limited economic development, weak healthcare infrastructure, poor governance, and low educational attainment (Rahman et al., 2022). Medically, leading causes of infant mortality include preterm birth complications, birth asphyxia, pneumonia, diarrheal diseases, malaria, and HIV/AIDS [5,6]. In addition, sociodemographic determinants such as maternal age, low birth weight, multiple births, place of delivery, inadequate breastfeeding practices, and short birth intervals further exacerbate infant mortality risks [7]. Reducing this burden requires comprehensive public health strategies, equitable access to quality healthcare, and targeted interventions to address both medical and structural determinants of infant mortality in the region.

Despite notable progress in reducing infant mortality, Ghana continues to face substantial challenges in meeting the Sustainable Development Goal (SDG) 3.2 target of reducing neonatal mortality to 12 and under-five mortality to 25 deaths per 1,000 live births by 2030 [8]. The country’s current infant mortality rate is approximately 28 deaths per 1,000 live births in 2023, reflecting improvement yet remaining above the global target [8]. Contributing factors include inadequate antenatal and postnatal care, limited breastfeeding support, shortages of skilled birth attendants, and persistent human resource constraints in the health sector [9]. Additional challenges such as poor maternal nutrition, infectious diseases, disparities in healthcare access across regions, insufficient infrastructure, limited availability of essential medicines, and entrenched cultural practices further exacerbate the burden [10]. These issues are especially pronounced in rural areas, where weak health infrastructure and inadequate transportation systems hinder timely access to essential maternal and neonatal care services [11,12].

Accurate estimation of child mortality rates is essential for informing effective policy design and targeted intervention strategies. Over time, a range of statistical approaches has been developed and refined to enhance the measurement and analysis of these rates. Comparative studies have evaluated the performance of various models to determine the most appropriate methods for identifying predictors of under-five mortality. For instance, an analysis of the 2016 Ethiopian Demographic and Health Survey (EDHS) data found the negative binomial model to provide the best fit for estimating child mortality predictors [13]. Likewise, in Greater Mexico City, Bayesian spatio-temporal modeling has been employed to examine the evolution of child mortality risk at the municipal level [14].

Several studies have employed hierarchical Bayesian modelling techniques to address over-dispersion due to varying population sizes across districts and to capture spatial autocorrelation commonly present in childhood mortality data. For instance, in Nigeria, district-level analyses have applied such models to account for spatial dependencies in mortality patterns [14]. In Ethiopia, Bayesian spatial analysis has been used to investigate geographic disparities and determinants of neonatal mortality [15]. A geostatistical survival model has also been utilized to quantify subnational variations in neonatal, infant, and under-five mortality rates across 99 low- and middle-income countries [16]. Schumacher et al. [17] introduced a flexible Bayesian framework to estimate age- and cause-specific child mortality using sample registration data. Furthermore, a Bayesian B-spline bias-reduction model was developed to generate global under-five mortality estimates while adjusting for systematic biases in data sources and providing uncertainty intervals for the period 1990–2019 [18]. In Ghana, Kwami et al. [19] modeled and mapped spatiotemporal trends in neonatal mortality risk using Bayesian hierarchical spatiotemporal models applied to five rounds of the Ghana Demographic and Health Surveys (GDHS) conducted between 1993 and 2014.

A study utilizing data from the 2014 Ghana Demographic and Health Survey (GDHS) applied geostatistical methods to examine the social and environmental determinants of under-five mortality [20]. This approach enabled the identification of mortality hotspots, which facilitated the targeting of interventions [20]. To estimate under-five mortality and its associated social and environmental risk factors at the district level in Ghana, indirect demographic methods alongside a Bayesian spatial model were employed [21]. A Bayesian logistic regression model was also used to assess the impact of selected risk factors on child mortality in Ghana [22]. Additionally, Kwami and colleagues proposed a method to model and map the spatiotemporal variations in the risk of neonatal mortality in Ghana using Bayesian Hierarchical Spatiotemporal models, utilizing data from the GDHS surveys conducted in 1993, 1998, 2003, 2008, and 2014 [19].

In Ghana, where the risk of child mortality varies markedly across regions and is influenced by economic disparities, urbanicity, and population mobility, rigorous statistical models that integrate socioeconomic and geographic information are essential for guiding targeted, evidence-based intervention. There is limited application of such models in the Ghana’s context [23,24]. Furthermore, advanced machine learning and interpretable modelling approaches such as Gradient Boosting combined with SHAP values [25] for explainable region-specific risk prediction remain underutilized.

In this study, child mortality incidence rates were computed for all regions and subsequently smoothed using Global and Local Empirical Bayesian approaches [26,27] to minimize random variation in areas with small populations. Spatial dependence and clustering were assessed using Global Moran’s I [27,28], LISA [27,29,30], and Getis–Ord Gi* statistics [31]. Spatial relationships between the risk of child mortality and selected socioeconomic and health-related determinants were examined using bivariate LISA [30]. Additionally, the relative contribution of predictors to child mortality risk was assessed using Gradient Boosting (GB), a nonparametric ensemble learning approach. Model interpretability was SHAP [25,32,33], enabling the derivation of global feature importance rankings and local explanation visualizations, including SHAP waterfall plots. Predictors identified as having strong associations with child mortality were subsequently incorporated into bivariate LISA analyses to examine their spatial relationships with child mortality risk. The BYM2 Bayesian spatial model was additionally employed to further examine the spatial distribution of child mortality risk in Ghana. Estimates from these methods were then visualized through maps to highlight regions with elevated child mortality risk in Ghana. The key hypotheses underpinning this study are: (1) child mortality risk is clustered among neighbouring regions, (2) child mortality risk varies across different regions, and (3) certain regional predictors are associated with either an increase or decrease in the risk of child mortality.

## 2. Design and methods

### 2.1. Study design

**Fig 1:**
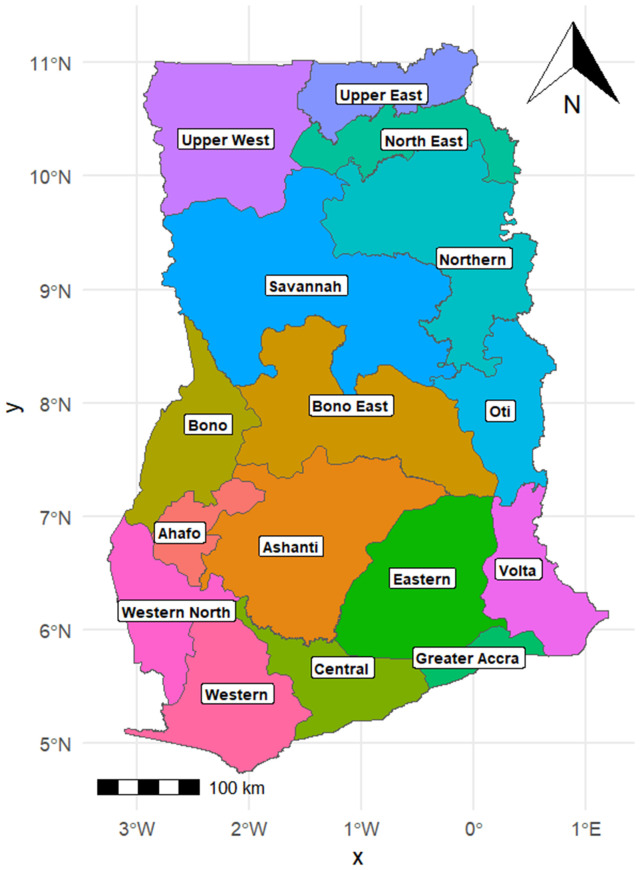
Map of Ghana showing 16 administrative regions.

An ecological study was carried out using real-world data on newly reported child deaths in Ghana in 2022, aimed at examining their association with socioeconomic and health-related factors. Ghana covers an area of approximately 238,533 km² and had an estimated population of 33.79 million in 2023, with a population density of 154 per km². The country is administratively divided into 16 regions (Fig 1).

### 2.2. Population and data sources

**Fig 2:**
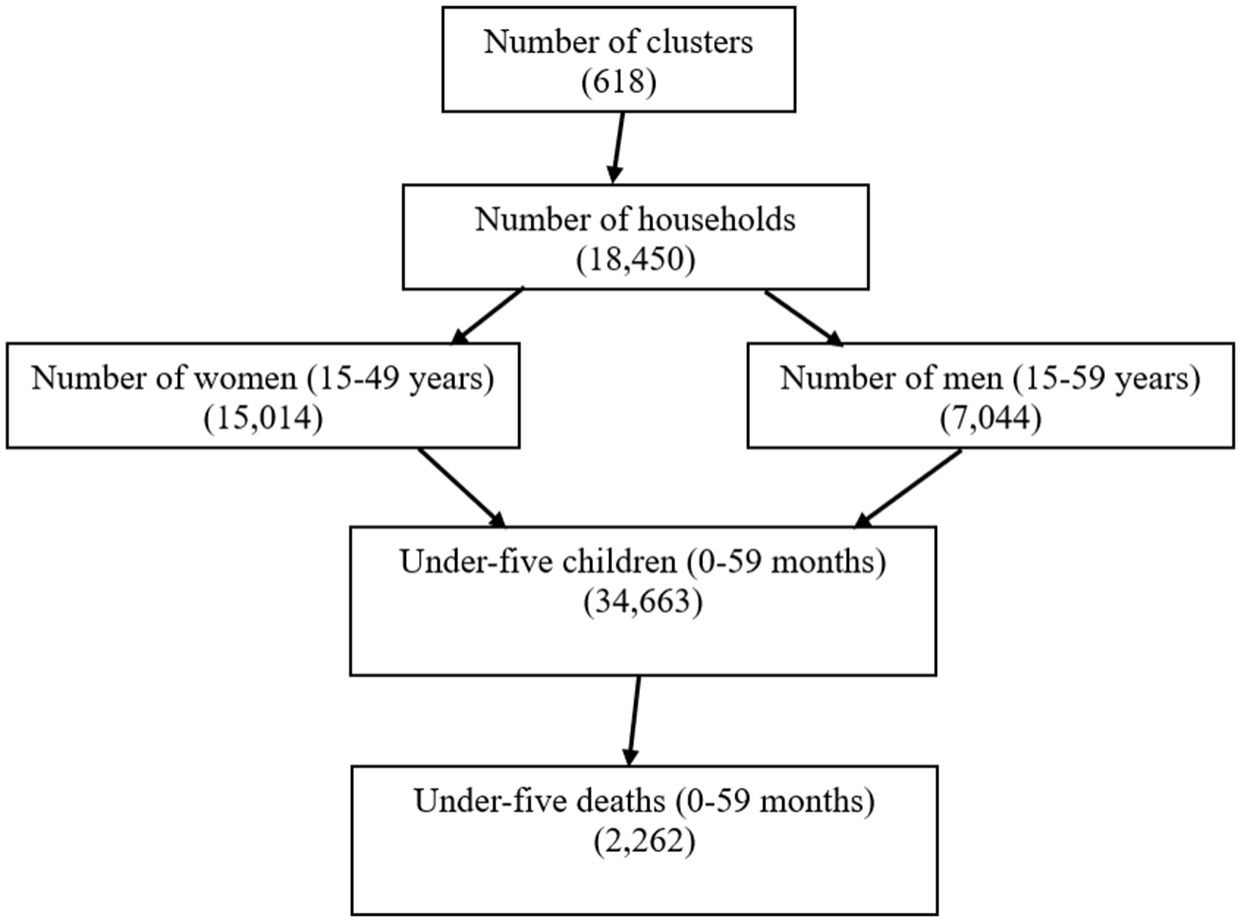
Flow-chart representing data collection procedure.

The data utilized in this study were sourced from the 2022 Ghana Demographic and Health Survey (GDHS 2022), publicly available at https://dhsprogram.com/methodology/survey/survey-display-598.cfm. Data on a total of 34,663 under-five children, collected as part of a nationally stratified, representative sample, with 2262 deaths across all the 16 regions, was used in this study. A total of 18,450 households were selected across 618 clusters, resulting in 15,014 interviewed women (aged 15–49 years) and 7,044 interviewed men (aged 15–59 years), with male respondents drawn from every second household (Fig 2). The sampling procedure employed in the GDHS 2022 followed a stratified two-stage cluster sampling design, ensuring national representation across urban and rural areas and each of the 16 regions for key DHS indicators.

First stage: A total of 618 clusters were selected using probability proportional to size (PPS) within urban and rural areas of each region. The final selection of clusters was conducted through systematic random sampling to ensure equal probability within designated strata. Second stage: A comprehensive household listing and map updating operation were carried out within the selected clusters to generate a complete sampling frame. A systematic random selection was then performed to identify the final household sample. This methodology was designed to ensure the representativeness and reliability of findings at both national and regional levels, facilitating robust statistical analysis of under-five mortality and associated factors.

**Ethics approval:** Access requires creating a user account and submitting a formal data request to MEASURE DHS, under the custodianship of the Institutional Review Board (IRB) of ICF International, for approval to access the relevant datasets used in this study.

### 2.3. Study variables

#### 2.3.1. Dependent variable.

The outcome variable is the number of children aged 0–59 months (under-five) who died per each of the 16 administrative regions in Ghana (Fig 1).

#### 2.3.2. Predictor variables.

This study examines various potential regional predictors of child mortality. These include percentage of women who used contraceptive pills, injectables contraceptives, attend antenatal care (ANC) at least 4 times, live in urban and rural areas, have abortion, delivered through caesarian section (CS), Gross national income (GNI) and consumer price index (CPI).

### 2.4. Spatial and statistical analysis

To investigate the spatial distribution of under-five mortality across Ghana, we first calculated crude death rates (CDR) for each region. Given the potential for random fluctuations, especially in regions with smaller populations, we applied smoothing techniques to stabilize these estimates. Specifically, we used two Empirical Bayes (EB) approaches [26,27]: the Global Empirical Bayesian (GEB) method, which adjusts each region’s CDR by weighting it against the national average, and the Local Empirical Bayesian (LEB) method, which smooths CDR based on the average rates of neighbouring regions.

To assess the presence and pattern of spatial clustering, we applied Global Moran’s I and the Getis-Ord Gi* statistics. Global Moran’s I measures the overall spatial autocorrelation across regions, with values ranging from −1 (indicating dispersion) to +1 (indicating clustering). A statistically significant Moran’s I (p < 0.05) suggests that under-five mortality is not randomly distributed but spatially structured [27,28].

To further identify localized clusters of high and low mortality, we conducted local spatial analyses using LISA, Getis–Ord Gi*, and the SaTScan spatial scan statistic [31]. The Getis–Ord Gi* analysis evaluates each region’s local spatial context by calculating z-scores and associated p-values, highlighting statistically significant hotspots (high mortality clusters) and coldspots (low mortality clusters) [29,30]. We also explored spatial correlations between under-five mortality and key regional indicators such as socioeconomic status and health conditions using bivariate spatial analyses. These included bivariate Global Moran’s I and bivariate LISA (BiLISA) [30], which allowed us to assess how mortality patterns align with potential predictors.

To examine the influence of these predictors on child mortality risk, we implemented gradient boosting (GB) [25,32,33], a nonparametric ensemble learning method. Variable importance rankings, and SHAP waterfall plots were used to interpret the impact of each factor on regional mortality. In this context, GB was included only as a supplementary exploratory method to assess possible nonlinear relationships and variable importance, and not as a substitute for the main regression-based spatial model. The BYM2 Bayesian spatial Poisson regression model was employed to characterize and quantify the spatial patterns of child mortality risk across Ghana. The Bayesian spatial BYM2 model served as the main analytical model in this study because it is appropriate for under-five mortality count data and is well suited for small-area ecological analysis [34]. The model assumes a Poisson likelihood, a standard choice in disease mapping for modelling observed counts relative to expected counts or population at risk. Furthermore, the BYM2 parameterization [35] accommodates both spatially structured and unstructured random effects, thereby accounting for geographic dependence and residual heterogeneity across regions. This feature improves the stability and interpretability of regional risk estimates [36].

The Bayesian framework also offers an important advantage in contexts where the number of observational units is limited. Through hierarchical shrinkage and prior-based regularization, Bayesian models can stabilize parameter estimation by borrowing strength across neighbouring areas and the broader data structure. This is especially valuable in small-area health studies, where sparse counts and limited sample size can otherwise produce unstable or highly variable estimates. Consequently, the BYM2 model provides a more robust inferential framework for regional under-five mortality analysis than purely non-spatial or highly data-demanding alternatives [37].

Analyses were performed using Python version 3.13 (https://www.python.org/downloads/) and R version 4.4.3 [38]. Statistical significance was assessed at the 5% level (p < 0.05).

## 3. Results

### 3.1. Crude death rate, global and local empirical Bayesian rates

**Fig 3:**
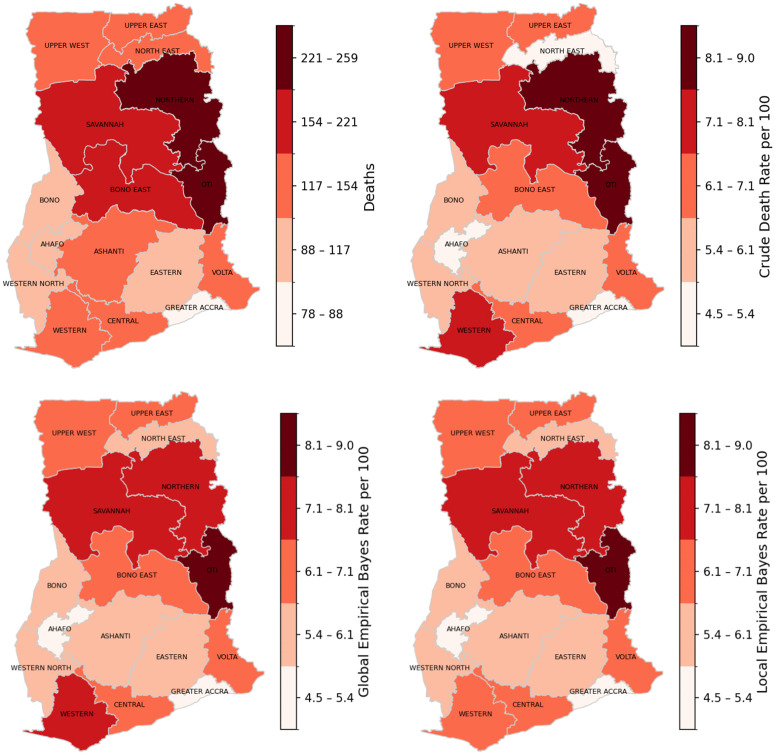
Spatial distribution of child mortality (top-left panel), crude death rate (tope-right panel), and smoothed rates by Global empirical Bayesian method (bottom-left panel) and Local empirical Bayesian method (bottom-right panel) per 100 live births per region of Ghana, 2022.

Regional analysis showed notable geographic disparities. Oti Region recorded the highest rates (CDR = 9.04; GEB = 8.57; LEB = 8.60 per 100 live births), all exceeding the national average, while Greater Accra (CDR = 4.49) and Ahafo (CDR = 4.79) recorded the lowest. The CDR was highest in Oti, followed by Northern (8.05) and Savannah (7.68) regions, whereas Ahafo and Greater Accra had the lowest values. For LEB, Oti (8.60), Northern (7.98), and Savannah (7.58) recorded the highest levels, compared with the lowest in Greater Accra (5.06) and Ahafo (5.29). Similarly, GEB was highest in Oti (8.57), Northern (7.82), and Savannah (7.48), but lowest in Greater Accra (4.99) and Ahafo (5.16). Overall, Oti, Northern, and Savannah consistently exhibited higher values for all three indicators, whereas Greater Accra and Ahafo were consistently among the lowest (Table 1 and Fig 3).

**Table 1:** Under-five mortality rates by region in 2022.

	Method		
Region	CDR/100	GEB/100	LEB/100
NORTHERN	8.05	7.82	7.98
NORTH EAST	5.38	5.58	5.78
SAVANNAH	7.68	7.48	7.58
BONO	5.39	5.68	5.87
AHAFO	4.79	5.16	5.29
BONO EAST	6.57	6.56	6.62
GREATER ACCRA	4.49	4.99	5.06
OTI	9.04	8.57	8.60
VOLTA	6.76	6.70	6.75
WESTERN	7.34	7.14	6.73
WESTERN NORTH	5.72	5.91	6.06
UPPER WEST	6.72	6.68	6.65
UPPER EAST	6.36	6.39	6.15
EASTERN	5.67	5.87	5.84
CENTRAL	6.78	6.73	6.47
ASHANTI	5.85	5.98	5.86

Across the three models (Table 2), mean estimates were similar (CDR = 6.41; GEB = 6.45; LEB = 6.45). The CDR showed greater variability (SD = 1.22; range = 4.49–9.04) than GEB (SD = 0.97; range = 4.99–8.57) or LEB (SD = 0.95; range = 5.06–8.60). Median values were comparable (CDR = 6.46; GEB = 6.48; LEB = 6.31), with a narrower interquartile range for LEB (0.88) relative to CDR (1.32) and GEB (1.01). Overall, the CDR exhibited greater regional dispersion, whereas GEB and LEB produced more stable estimates, less sensitive to random fluctuations, and therefore better suited for spatial comparison and mappingg [27].

**Table 2:** Summary statistics of prevalence of child mortality in 2022.

Estimate	Mean (SD)/100 live births	Median (IQR)/ 100 live births	Range (Min, Max)/ 100 live births
CDR	6.41 (1.22)	6.46 (1.32)	(4.49, 9.04)
GEB	6.45 (0.97)	6.48 (1.01)	(4.99, 8.57)
LEB	6.45 (0.95)	6.31 (0.88)	(5.06, 8.60)

Child mortality rates, displayed in Fig 3, show a distinct spatial pattern across Ghana, with the highest crude death rates observed in the northern regions, particularly Oti, Northern, and Savannah, while lower rates occur in southern regions such as Greater Accra and Ahafo; however, the Western Region records the highest rate in the south. These crude death rates largely reflect raw death counts, which are influenced by population size and random variation. After applying Global Empirical Bayes (GEB) smoothing, extreme values, especially in areas with small populations, are moderated, resulting in a more balanced spatial distribution while retaining the north–south mortality gradient. The Local Empirical Bayes (LEB) approach further stabilizes rates by reducing random noise in low-population regions and highlights consistent clusters of high mortality in Northern, Oti, and Savannah regions. Overall, all three approaches consistently reveal a pronounced north–south divide in child mortality, with the highest rates concentrated in northern Ghana.

These findings underscore the need for targeted spatial analyses, such as Moran’s I or spatial scan statistics, to formally detect significant clusters and guide region-specific public health interventions.

### 3.2. Spatial autocorrelation and local clustering of child mortality in Ghana

**Fig 4:**
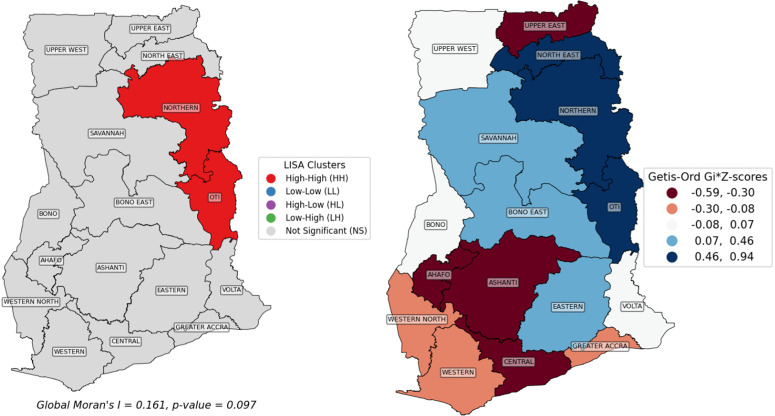
Spatial clustering of child mortality in Ghana per region in 2022, based on local spatial correlation–LISA (left panel) and Getis-Ord Gi* statistic (right panel).

Although the Global Moran’s I was not statistically significant (I = 0.161, p = 0.097), indicating no strong nationwide spatial autocorrelation, the LISA analysis still identified significant localized clusters. This is methodologically plausible because global and local spatial statistics capture different aspects of spatial patterning. Also, this situation is particularly plausible in studies with a small number of spatial units, such as the 16 administrative regions used in the present analysis, where global tests may have limited statistical power to detect modest or spatially concentrated patterns. Global Moran’s I reflects the overall spatial structure across all regions, whereas LISA detects specific local pockets of clustering. LISA revealed sub-regional clusters and spatial outliers, highlighting geographically confined areas where under-five mortality risk is spatially correlated. These local patterns reflect heterogeneity in risk that is not captured by the global statistic and underscore the importance of distinguishing between national-level spatial structure and localized spatial processes. The LISA map identified significant high–high clusters in the Northern and Oti regions, where elevated mortality rates are surrounded by similarly high-risk neighbours such as Volta, Savannah, and Bono East (Fig 4, **left panel**). The Getis-Ord Gi* analysis (Fig 4, right panel and Table 3) corroborated these findings, detecting statistically significant hotspots in Northern (Z = 0.94, p = 0.043) and Oti (Z = 0.71, p = 0.022), while other regions, including North East (Z = 0.61, p = 0.084) and Savannah (Z = 0.46, p = 0.095), did not meet the significance threshold. No coldspots patterns were identified. Overall, these results indicate that although child mortality does not exhibit strong national-scale clustering, localized hotspots persist in Northern and Oti regions, underscoring the need for geographically targeted interventions to reduce child mortality in these high-risk areas. These local clusters should therefore be interpreted cautiously as subnational spatial concentrations rather than evidence of uniform national clustering. Also, although Savannah Region had relatively high CDR and LEB values, it did not emerge as a statistically significant hotspot in the Getis-Ord Gi* or LISA analysis. This is because, the local spatial significance depends not only on the value observed in a single region, but also on the values of neighbouring regions and the overall local spatial configuration. A region with a high mortality rate may therefore fail to appear as a hotspot if surrounding areas do not exhibit a sufficiently similar concentration of high values. This suggests that Savannah’s elevated mortality burden may be epidemiologically important, even though it did not form a statistically significant localized spatial cluster under the methods used.

**Table 3:** Gi* Z-score interpretation summary.

Region	Z-score	P-value	Interpretation
Northern	0.94	0.043	Significant (Hotspot, 95%CI)
Oti	0.71	0.022	Significant (Hotspot, 95%CI)
North East	0.61	0.084	Not significant
Savannah	0.46	0.095	Not significant
Bono East	0.27	0.255	Not significant
Eastern	0.10	0.380	Not significant
Upper West	0.07	0.449	Not significant
Volta	0.01	0.471	Not significant
Bono	-0.02	0.486	Not significant
Western	-0.08	0.500	Not significant
Western North	-0.10	0.401	Not significant
Greater Accra	-0.12	0.449	Not significant
Upper East	-0.30	0.292	Not significant
Central	-0.47	0.107	Not significant
Ashanti	-0.52	0.050	Not significant
Ahafo	-0.59	0.325	Not significant

### 3.3. Spatial clustering of child mortality in Ghana using SaTScan

**Fig 5:**
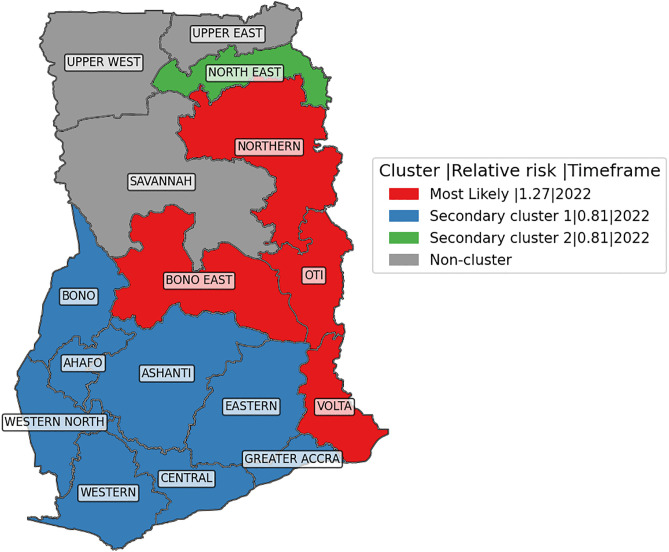
Spatial clusters of child mortality in Ghana, 2022.

The SaTScan spatial analysis (Table 4 and Fig 5) identified one most likely cluster and two additional clusters of under-five mortality across Ghana in 2022, based on the discrete Poisson model. The most likely (primary) cluster comprised the Northern, Bono East, Oti, and Volta regions. This high-risk cluster exhibited a relative risk (RR) of 1.27 and a p-value < 0.001, indicating that the risk of under-five mortality was 27% higher within this cluster compared to areas outside it. This suggests a significantly elevated burden of mortality concentrated in these regions.

**Table 4:** SaTScan cluster analysis results based on regional child mortality Data (2022).

Cluster Type	Regions	Cluster Population	Observed Cases	Expected Cases	CDR/100	LLR	RR	P-value
Most likely cluster (4): High Rate	Northern, Bono East, Volta, Oti	9,735	751	635.28	7.71	14.170	1.27	0.001
Secondary Cluster 1 (8): Low Rate	Western, Western North, Central, Ahafo, Ashanti, Eastern, Greater Accra, Bono	15,251	879	995.23	5.76	12.245	0.81	0.001
Secondary Cluster 2 (1): Low Rate	North East	2,713	146	177.04	5.38	3.126	0.81	0.352

RR: relative risk; LLR: log-likelihood; CDR/100: crude incidence rate per 100.

The first secondary cluster included Western, Western North, Central, Ahafo, Ashanti, Eastern, Greater Accra, and Bono regions. This low-risk cluster had an RR of 0.81 and a p-value of 0.001, reflecting a 19% lower risk of under-five mortality relative to regions outside the cluster. The second secondary cluster was limited to the North East region, which also showed a lower risk with an RR of 0.81, although the result was not statistically significant (p = 0.352). Despite being geographically close to the high-risk primary cluster, this region showed a comparatively lower mortality rate. Regions not included in any statistically significant cluster such as Upper West, Upper East, Savannah, and others were classified as non-clustered, indicating average or spatially heterogeneous mortality risks.

### 3.4. Bivariate Moran’s I analysis

**Fig 6:**
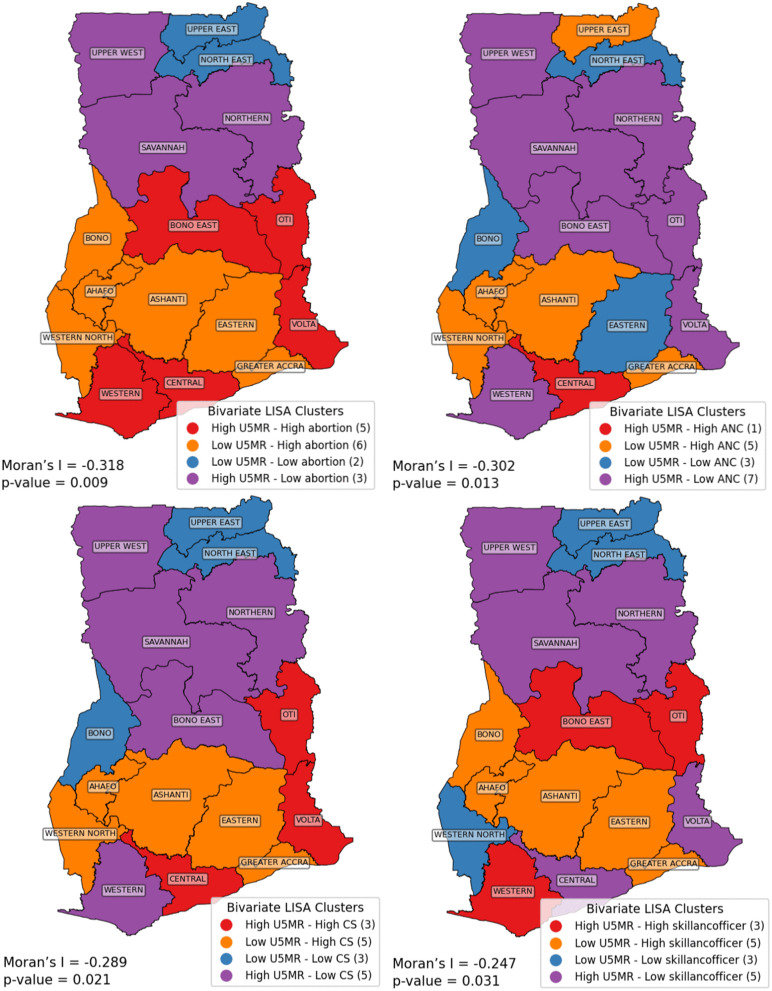
Bivariate analysis (LISA) of child mortality and abortions rare, ANC coverage, CS rate, and skilled birth attendance with statistically significant spatial autocorrelation.

We examined the spatial relationships between under-five mortality rate (U5MR) and some maternal health indicators; abortion rate, antenatal care (ANC) coverage, caesarean section (CS) rate, and skilled birth attendance using bivariate LISA cluster analysis across regions in Ghana. The resulting cluster maps and associated global Moran’s I statistics (Fig 6) reveal significant negative spatial associations in all cases, indicating that regions with high U5MR often coincide with poor maternal health service coverage, and vice versa.

The spatial association between U5MR and abortion rate was significantly negative (Moran’s I = -0.318, *p* = 0.009), suggesting spatial clustering of high U5MR with low abortion rates, and low U5MR with high abortion rates. High-high clusters were observed in Volta, Oti, Bono East, Central and Western regions while the northern corridor; including Northern, Savannah and Upper West formed a high U5MR–low abortion cluster. These patterns highlight spatial inequalities where regions with elevated child mortality may also have unmet reproductive health needs.

A significant negative spatial correlation was also found between U5MR and ANC coverage (Moran’s I = -0.302, *p* = 0.013). High U5MR–low ANC clusters were prominent in northern belt, pointing to deficiencies in maternal health services. A notable exception was the Central region, which showed a high-high cluster (high U5MR and high ANC), possibly indicating poor service quality or delayed initiation of ANC despite high coverage.

The spatial association between U5MR and CS rate was significantly negative (Moran’s I = -0.289, *p* = 0.021). Regions in the north, particularly Upper West, Savannah, Bono East, and Northern, and Western region in the South, showed high U5MR coupled with low CS rates. In contrast, southern regions like Ashanti, Eastern, Ahafo, Western North, and Greater Accra formed low U5MR-high CS clusters, suggesting better emergency obstetric care in those areas. The Volta, Oti, and Central regions, however, exhibited high U5MR despite high CS rates, indicating potential issues with service timeliness or quality.

The association between U5MR and skilled birth attendance was weaker but still statistically significant (Moran’s I = -0.247, *p* = 0.031). High U5MR–low skilled attendance clusters were again concentrated in the northern belt, reinforcing the persistent disparity in maternal health access. Western, Oti and Bono East regions emerged as high U5MR–high skilled attendance clusters, again raising concerns about the effectiveness and quality of skilled birth services.

BiLISA cluster maps for under-five mortality rate (U5MR) and the other variables are presented in S1 and S2 Appendix. Unlike the previous maps (in Fig 4), these exhibits weaker or statistically non-significant spatial associations, yet they still provide valuable insights into regional clustering patterns.

The association between U5MR and injectable contraceptive use showed a weak negative spatial correlation (Moran’s I = -0.226, *p* = 0.077). High U5MR–low injectable clusters were concentrated in the northern regions, while low U5MR–high injectable clusters were observed in North East, Ashanti, Western North, Ahafo, and Upper East. High-high cluster was observed in Western region. Similarly, U5MR and smoking prevalence exhibited a weak and non-significant negative association (Moran’s I = -0.152, *p* = 0.157). High U5MR–high smoking clusters were located in Upper West, Oti, and Savannah, while Greater Accra and Ashanti displayed low U5MR–low smoking patterns.

The spatial relationship between U5MR and oral contraceptive pill use (Moran’s I = -0.130, *p* = 0.175) showed high U5MR–high pill use clustering across the northern and middle belt, with low U5MR–low pill uses in Greater Accra, Upper East, and North East. Also, U5MR showed a weakly positive spatial association with rural residence (Moran’s I = 0.185, *p* = 0.082). High U5MR–high rural clusters dominated northern and western Ghana, while low U5MR–low rural clusters were concentrated in more urbanized regions such as Ashanti and Greater Accra.

Neither relationship was statistically significant at the global level (GNI: Moran’s I = 0.158, *p* = 0.145; CPI: Moran’s I = 0.075, *p* = 0.365), but important local spatial clusters were observed. For U5MR and GNI, High U5MR–High GNI clusters occurred in Northern, Savannah, Upper West, and Oti, suggesting that higher income alone does not guarantee better child health outcomes. Low U5MR–High GNI clusters in Bono, Ashanti, North East, and Upper East showed alignment between wealth and improved survival. Some regions like Greater Accra and Ahafo showed low U5MR despite low GNI, possibly reflecting effective health systems. In the U5MR–CPI analysis, High U5MR–High CPI clusters appeared in Northern, Bono East, Central, Western, and Upper West, indicating potential governance and service delivery challenges. Low U5MR–High CPI clusters were found in Bono and Ashanti, while Ahafo and Upper East formed Low U5MR–Low CPI clusters. High U5MR–Low CPI regions, such as Savannah and Oti, suggest that low perceived corruption alone is insufficient to reduce child mortality.

### 3.5. Gradient boosting

#### 3.5.1. Variable importance and partial dependence plots.

**Fig 7:**
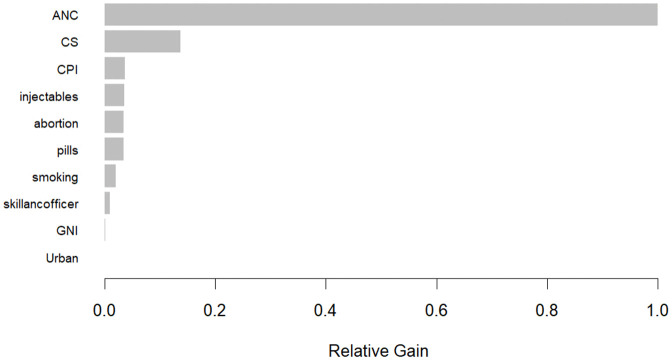
Gradient boosting model: variables importance plot.

To further assess the impact of the covariates on child mortality, a Gradient Boosting (GB) model was applied. The resulting variable importance, illustrated in Fig 7, was evaluated using the gain metric. The variable importance results based on **Gain** indicate that antenatal care (ANC) coverage is the most influential predictor, accounting for approximately **76%** of the total model gain, highlighting its dominant role in improving model performance. Caesarean section (CS) use follows but contributes much less, with around **10%** of the gain. Consumer Price Index (CPI), injectables, abortion, and pills each contribute modestly (approximately **2–3%** each), suggesting limited but notable influence. Smoking and skilled ANC officer availability show very minor contributions (<2%), while gross national income (GNI) and urban residence have near-zero gain values, indicating negligible impact on prediction accuracy. Overall, ANC coverage overwhelmingly drives the model’s predictive ability, with all other variables providing relatively small incremental improvements. The importance of ANC and CS in predicting child mortality was further supported by the Moran’s I analysis.

#### 3.5.2. SHapley additive exPlanations (SHAP) waterfall plots.

**Fig 8:**
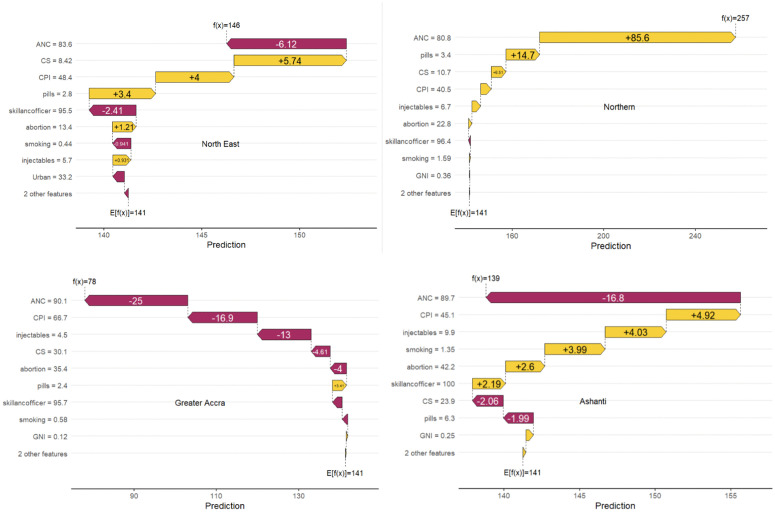
SHAP waterfall plot: North East (top-left panel), Northern (top-right panel), Greater Accra (bottom-left panel), and Ashanti (bottom-right panel) regions.

To further understand the contribution of individual variables to regional predictions generated by our machine learning model, we employed SHAP values, visualized through waterfall plots for four selected regions: North East, Northern, Greater Accra, and Ashanti (**Fig 8**). Similar plots (S3–S5 Appendix) for the remaining regions are presented as supplementary materials.

The SHAP waterfall plots (Fig 8) identified key regional drivers of child mortality risk. The predicted values represent model-estimated under-five mortality rates per 1,000 live births. For example, in the SHAP waterfall plot for the North East region, the baseline expectation is E[f(x)] = 141, which corresponds to the average predicted under-five mortality rate across all regions. The model then sequentially adds or subtracts contributions from each predictor. ANC coverage (-6.12) reduces the predicted mortality relative to the baseline, while caesarean section coverage (+5.74), contraceptive prevalence index (+4), and pills use (+3.4) increase it. The final predicted value of f(x) = 146 represents the estimated under-five mortality rate for the North East region based on its observed covariate profile. That is, in **North East**, the predicted risk (146) was slightly above the national baseline (141), driven by higher caesarean section coverage (+5.74) and pill use (+3.4), though this was partially offset by reductions from antenatal care (ANC) coverage (−6.12) and skilled ANC officer availability (−2.41). **Northern** region showed a much higher under-five mortality rate (257), primarily due to an unusually large positive contribution from ANC coverage (+85.6), suggesting possible unmeasured risk factors despite apparent service availability. The positive SHAP contribution of ANC coverage in the Northern Region was an unexpected finding and should be interpreted cautiously. In this analysis, positive SHAP values indicate that a predictor increases the model-predicted under-five mortality, while negative values indicate a lowering effect relative to the baseline prediction relative to the baseline prediction. This does not imply a causal harmful effect of ANC. Instead, the result may reflect a quality coverage paradox, whereby higher reported ANC utilization does not necessarily translate into improved child survival if the quality, timing, or continuity of care is inadequate. It may also reflect model instability or overfitting, given the very small number of regions included in the machine learning analysis. Accordingly, this finding should be interpreted as exploratory and hypothesis-generating, rather than conclusive. In **Greater Accra**, the predicted risk was the lowest (78), largely reduced by ANC coverage (−25), CPI (−16.9), injectable contraceptive use (−13), and CS coverage (−4.61), indicating strong healthcare access and favorable economic conditions. **Ashanti** had a predicted risk (139) close to the baseline, with reductions from ANC (−16.8) and skilled staff (−2.19) balanced by risk increases from injectables (+4.03), smoking (+3.99), and abortion (+2.6). Overall, ANC coverage consistently influenced child mortality risk, while economic conditions and contraceptive patterns contributed to regional variation.

The analysis of SHAP waterfall plots (S3 Appendix) across Ahafo, Bono, Savannah, and Volta regions highlight ANC coverage as the most influential factor affecting model predictions. Regions with low ANC coverage, such as Ahafo (f(x) = 97.4) and Bono (f(x) = 88.2), had significantly lower predicted outcomes, with ANC reducing predictions by over 20 units in both cases. In contrast, Savannah recorded a much higher prediction (f(x) = 207), primarily driven by a strong positive contribution from ANC (+67) and caesarean section rates (+6.25). Volta had a moderate prediction (f(x) = 117), with ANC again contributing negatively (-23.7), though gains were observed from CPI (+5.52) and contraceptive use.

The SHAP waterfall plots (S4 Appendix) reveal that **ANC coverage** is the most influential factor driving regional differences in predicted health outcomes. **Oti** had the highest predicted value (221), largely due to strong ANC coverage (+77.8) and CS use (+6.67). **Eastern** recorded the lowest prediction (105), driven by negative contributions from ANC (−18), CPI (−17.2), and CS (−3.46), indicating poor service access and economic conditions. **Bono East** (153) and **Central** (142) had predictions near or slightly above the national baseline, with positive contributions from CPI, contraceptive use, and CS, but were offset by low ANC in Central (−17.5) and modest negative effects in Bono East. The SHAP analysis (S5 Appendix) reveals that **ANC coverage** is the most consistent negative driver of predicted health outcomes across all four regions. **Upper East** (f(x) = 136) had a slightly below-average prediction due to low ANC coverage (−22.4), though positive contributions from CPI, contraceptive use, and abortion care partially offset this. **Upper West** (f(x) = 143) performed slightly above the baseline, with strong positive influences from CS (+8.19), CPI (+6.34), and contraceptive use, outweighing the negative impact of ANC. **Western** (f(x) = 127) showed a lower prediction driven by poor ANC (−17.6) and CS coverage, despite support from injectables and CPI. **Western North** (f(x) = 103) had the lowest score, with widespread negative contributions from ANC, CS, abortion, injectables, and CPI.

### 3.6. Spatial distribution of relative risk of child mortality

#### 3.6.1. Bayesian Poisson and Bayesian Negative Binomial spatial models’ comparison.

In this section, we compared the Bayesian spatial Poisson and Bayesian spatial Negative Binomial models, both fitted with BYM2 random effects (spatially structured and unstructured) [35], to identify the model that best captured the spatial variation in the outcome. Model performance was assessed using Deviance Information Criterion (DIC) [39], Watanabe-Akaike Information Criterion (WAIC) [40], effective number of parameters (pD), and Log Pseudo Marginal Likelihood (LPML), with lower DIC/WAIC values and higher LPML values indicating better performance. These performance metrics are presented in Table 5.

**Table 5:** Comparison of Bayesian spatial Poisson and Bayesian spatial Negative Binomial models with BYM2 random effects using DIC, WAIC, and LPML.

Indicator	Bayesian spatial Poisson model with BYM/BYM2 random effects	Bayesian spatial Negative Binomial model with BYM/BYM2 random effect
DIC (pD)	138.89 (15.00)	155.20 (14.18)
WAIC (pD)	135.14 (8.10)	153.37 (9.52)
COP: Log pseudo marginal likelihood (LPML)	-95.53	-161.71

The Bayesian spatial Poisson model had lower DIC (138.89) and WAIC (135.14) values compared with the Negative Binomial model (155.20 and 153.37, respectively). It also had a higher LPML (-95.53 vs -161.71), indicating better predictive ability. The superior performance of the Bayesian spatial Poisson model suggests that the variability in the outcome was adequately captured by the spatially structured and unstructured random effects, making the additional overdispersion component of the Negative Binomial model unnecessary.

#### 3.6.2. Results from Bayesian Poisson spatial BYM2 model.

In this section, three Bayesian spatial models based on the BYM2 specification were fitted to examine the factors associated with under-five mortality. BYM2 Model I incorporated all candidate predictors considered relevant to under-five mortality. BYM2 Model II included only the subset of variables (ANC, CS, CPI, Injectables, Pills) identified by the gradient boosting (GB) model as the most influential predictors. In contrast, BYM2 Model III was specified using only the two top-ranked predictors, namely ANC and CS. The comparative performance of these three models was assessed using the model fit and predictive performance criteria summarized in Table 5. The parameter estimates and corresponding results for the fitted models are presented in Table 6.

**Table 6:** Comparison of BYM2 Model I–III based on model fit and predictive performance criteria (DIC, WAIC, and LPML) for under-five mortality.

Indicator	BYM2 Model I	BYM2 Model II	BYM2 Model III
DIC (pD)	138.89 (15.00)	137.85 (14.12)	136.34 (12.55)
WAIC (pD)	135.14 (8.10)	134.59 (7.86)	134.32 (7.67)
LPML	-95.53	-86.71	-77.27

Model comparison based on DIC, WAIC, and LPML showed that BYM2 Model III performed best among the three fitted models. It had the lowest DIC (136.34) and WAIC (134.32), as well as the highest LPML (-77.27), indicating superior fit and predictive performance relative to BYM2 Model I and BYM2 Model II. These results suggest that the model including only ANC and CS provided the most parsimonious and best-performing explanation of under-five mortality.

The results from the BYM2 Model III with ANC and CS covariates are presented in Table 7 with convergence diagnostics presented in Fig 10 showed that parameter estimates have converged. The intercept had a posterior mean of 3.26 with a 95% CrI: 0.60-17.90, suggesting that the baseline risk of under-five mortality was elevated when all covariates were held at their reference or baseline levels. However, the wide credible interval indicates considerable uncertainty around this baseline estimate. For ANC, the posterior mean relative risk was 0.99 (95% CrI: 0.97–1.01). This suggests that ANC coverage was associated with a slight reduction in the risk of under-five mortality. Specifically, a one-unit increase in ANC was associated with approximately a 1.2% lower risk of under-five mortality (1-0.9876)×100(1-0.9876) × 100(1-0.9876)×100. However, because the 95% credible interval includes 1, there is no strong statistical evidence that ANC had a meaningful independent effect on under-five mortality in the fitted spatial model. Similarly, CS had a posterior mean relative risk of 0.99 (95% CrI: 0.97–1.02), indicating a very small inverse association with under-five mortality. In practical terms, a one-unit increase in CS was associated with about a 0.6% reduction in the risk of under-five mortality (1-0.9940)×100(1-0.9940) × 100(1-0.9940)×100. Nonetheless, the 95% credible interval also crosses 1, suggesting that this effect was not statistically important after accounting for spatial variation and uncertainty.

**Table 7:** Posterior parameter estimates expressed as relative risks (RR) with corresponding standard errors and 95% credible intervals for the best-fitting BYM2 model.

Variable	Estimate (RR)	Standard Error (SE)	95% Credible Interval (CrI)
Intercept	3.26	2.38	0.60 – 17.90
ANC	0.99	1.01	0.97 – 1.01
CS	0.99	1.01	0.97 – 1.02

**Fig 9:**
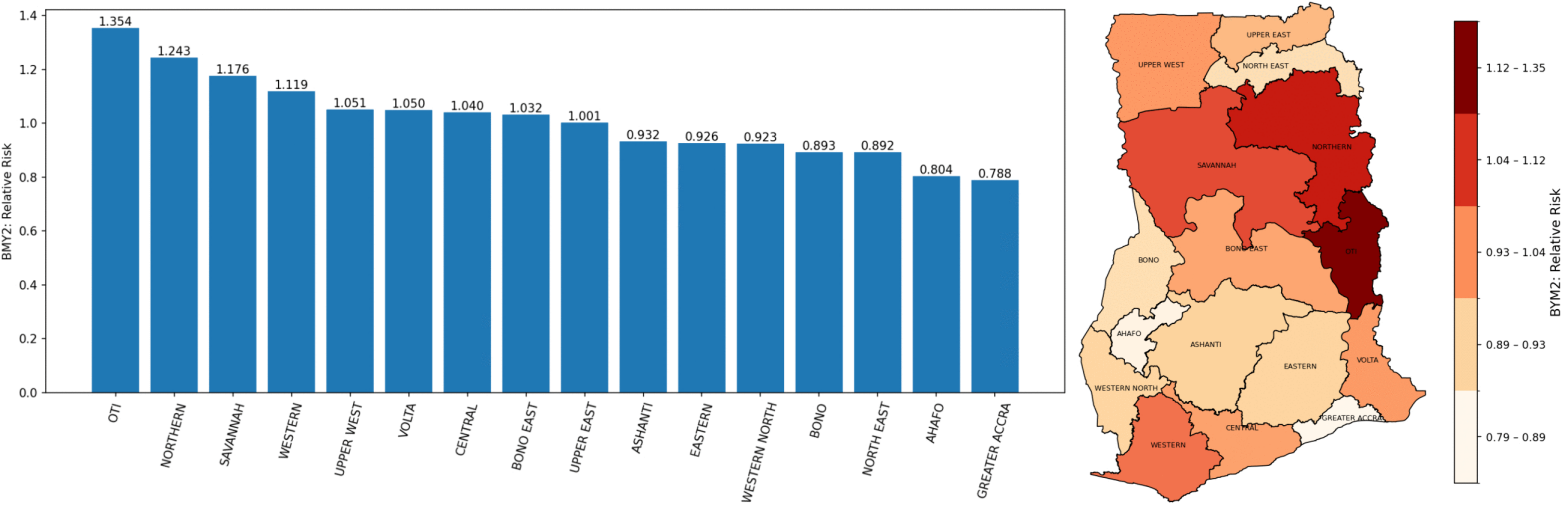
BYM2 model: Bar graph (left panel) and relative risk map (right panel) representing regional relative risk of child mortality in Ghana.

**Fig 10:**
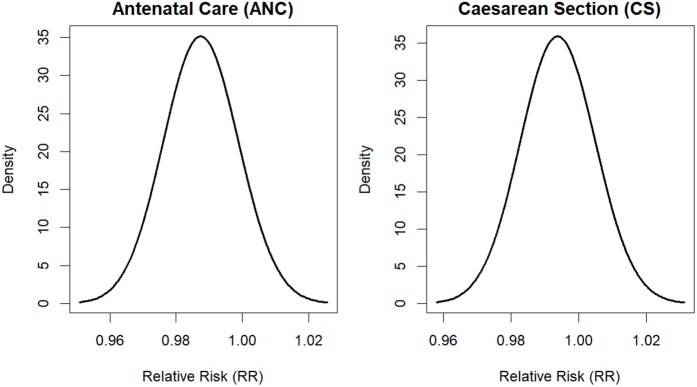
Posterior convergence diagnostics for the selected BYM2 Model III.

The relative risk of child mortality, for each region, was estimated using the BYM2 Model III. The left panel of Fig 9 and the right panel of Fig 9 present the relative risk of child mortality using a bar graph and a relative risk map, respectively. A relative risk greater than 1 indicates an increased risk, a value less than 1 signifies a reduced risk, and a value of 1 represents no significant risk difference. The results from the BYM2 model agree with the finding from the spatial models used.

## 4. Discussion

In this study, investigated the spatial distribution of under-five (U5MR) in Ghana. Our analysis confirms that northern Ghana, including the Oti, Northern, and Savannah regions, experiences substantially higher under-five mortality rates (U5MR). This finding aligns with earlier demographic studies that documented persistent regional and rural–urban disparities in child mortality across Ghana, especially in favor of Greater Accra [41,42]. The use of Empirical Bayes smoothing in our study, which reduced rate variability while preserving geographic patterns, adds methodological robustness compared to traditional CDR analyses. Spatial clustering and SaTScan findings, highlighting mortality hotspots in Northern belt, underscore longstanding structural inequities [37,38]. Consistent with earlier GDHS-based analyses and geostatistical studies [19,43], our results reaffirm a persistent north–south gradient in under-five mortality in Ghana, with substantially higher risks in the northern belt compared with southern regions such as Greater Accra and Ahafo. Previous analyses [19,43] using pooled GDHS data from 1993-2014 and the 2014 GDHS typically identified Northern, Upper East, and Upper West as broad high-risk zones, reflecting long-standing structural disadvantages related to poverty, maternal education, health service access, and environmental conditions. Our results corroborate spatially stratified findings from other Ghanaian studies identifying high-risk areas tied to service access deficits and rural poverty [42,44]. However, our 2022 findings corroborate this general pattern but reveal important spatial refinements.

First, unlike earlier surveys [19] that often highlighted the Upper East and Upper West as dominant hotspots, our study identifies Oti and Northern regions as the most consistent and statistically significant hotspots across multiple methods (CDR, GEB, LEB, LISA, Getis-Ord Gi*, SaTScan and BYM2). The emergence of Oti as a high-risk region represents a notable shift from earlier GDHS periods, during which Oti was not separately delineated or did not consistently emerge as a hotspot. This suggests a reconfiguration of spatial risk following administrative boundary changes and evolving regional inequalities after 2014.

Second, while earlier geostatistical analyses [43] tended to report broader contiguous northern clusters, our use of complementary local (LISA, Gi*) and scan-statistic (SaTScan) approaches reveals more localized and heterogeneous clustering. For example, Savannah often grouped with other northern regions in earlier work shows elevated rates descriptively but does not consistently meet statistical significance as a hotspot in 2022, whereas Northern and Oti do. This distinction highlights that not all northern regions currently experience the same level of excess risk, an insight that was less apparent in earlier national or regional-level analyses.

Third, our SaTScan results provide new evidence on both high- and low-risk clusters. The identification of a primary high-risk cluster spanning Northern, Oti, Bono East, and Volta regions (RR = 1.27, p < 0.001) contrasts with earlier GDHS analyses that largely confined elevated risk to the far north. At the same time, the detection of a statistically significant low-risk cluster covering much of southern Ghana (including Greater Accra, Ashanti, and Eastern regions) reinforces and quantifies the protective advantage of these regions in 2022, extending prior descriptive findings.

Overall, compared with earlier GDHS-based studies (1993–2014 and 2014 GDHS) [19], our analysis demonstrates both continuity and change: continuity in the enduring north–south divide, and novelty in the identification of Oti and Northern as the most robust contemporary hotspots, alongside a more nuanced, localized clustering pattern. By leveraging updated 2022 data and multiple spatial techniques, our study provides a more current and policy-relevant picture of under-five mortality risk in Ghana, strengthening the validity and added value of our findings relative to prior work.

Antenatal care (ANC) emerged as the most influential predictor of U5MR, echoing broader public health evidence that ANC quality and coverage critically affect child survival outcomes [45]. However, in regions with high ANC coverage yet persistent mortality, our findings suggest quality deficiencies as documented in Northern Ghana may limit ANC protective impact [46]. While wealth and governance indicators had weaker associations with mortality, our local-level clusters suggest income alone does not assure lower child mortality, supporting growing literature on the limits of economic gains when health systems remain inequitable [44,47]. Evidence from Ghana suggests that high ANC attendance does not necessarily translate into effective care, as substantial gaps persist in the content and quality of ANC services delivered. Studies have documented incomplete provision of essential ANC components, including blood pressure measurement, iron–folate supplementation, tetanus toxoid vaccination, and early detection of pregnancy complications, even in settings with high coverage [48,49]. Health system factors, including facility readiness, staffing, and referral capacity, have also been shown to vary spatially in Ghana and may further contribute to the observed discordance between ANC coverage and under-five mortality risk [50]. These findings suggest that ANC coverage alone may be insufficient to reduce child mortality in the absence of consistent, high-quality service delivery and supportive health system infrastructure. This points to the urgency for place-sensitive, multisectoral strategies in high-risk regions.

First, the consistent identification of Northern and Oti as high-risk clusters across crude, smoothed, and spatial scan analyses suggests the need to prioritize strengthening primary maternal, newborn, and child health (MNCH) services in these regions. This includes expanding the availability and functionality of Community-based Health Planning and Services (CHPS) compounds, ensuring consistent staffing with skilled midwives and community health nurses, and improving referral linkages to district hospitals for obstetric and neonatal emergencies. Earlier Ghanaian studies (e.g., Aheto et al. [43],) have shown that gaps in skilled delivery and postnatal care contribute substantially to child mortality in northern Ghana; our results indicate that these gaps remain geographically concentrated.

Second, the presence of localized hotspots rather than uniform national clustering supports prioritising targeted antenatal, delivery, and postnatal interventions in hotspot districts within Northern and Oti regions. Programmes such as early and frequent antenatal care attendance, iron-folate supplementation, malaria prevention in pregnancy (IPTp), and strengthened postnatal follow-up in the first 28 days of life are particularly relevant, given that neonatal deaths contribute a large share of under-five mortality in these settings.

Third, the north-south gradient observed after empirical Bayes smoothing points to the importance of child survival interventions beyond the health facility, especially in rural and sparsely populated areas. These include scaling up routine immunization outreach, integrated management of childhood illnesses (IMCI), and community-based management of malaria, pneumonia, and diarrhoeal diseases. Such interventions are especially critical in Northern and Oti regions, where distance to facilities and population dispersion increase vulnerability to preventable child deaths.

Fourth, the identification of a high-risk SaTScan cluster spanning Northern, Oti, Bono East, and Volta suggests a need for cross-regional and corridor-based strategies, rather than isolated regional programmes. Mobile outreach services, harmonized supply chains for essential medicines, and coordinated supervision across neighbouring regions could help address spill-over risks in border districts that share similar socio-ecological and health system constraints.

Finally, given that earlier Ghanaian and West African studies link child mortality in the northern belt to broader structural factors, our findings support prioritising nutrition-sensitive and social protection programmes alongside health interventions. These include maternal nutrition support, promotion of exclusive breastfeeding, community-based management of acute malnutrition, and integration with poverty-alleviation and female education initiatives. The persistence of hotspots in 2022 indicates that health-sector interventions alone may be insufficient without addressing these underlying determinants.

Overall, our spatially explicit results argue for a shift from uniform national programming toward geographically targeted MNCH strategies, with intensified investment in Northern and Oti regions. Such prioritization would align limited resources with the areas of greatest excess risk and is likely to yield the largest reductions in under-five mortality in Ghana.

The findings from BYM2 model, which retained antenatal care (ANC) and caesarean section (CS) as covariates, indicate that both variables were associated with slight reductions in the relative risk of under-five mortality, although neither showed strong statistical evidence after accounting for spatial dependence and uncertainty. The inverse association observed for ANC is biologically and programmatically plausible, given its established role in improving maternal health monitoring, facilitating early detection and management of pregnancy-related complications, and strengthening linkage to essential maternal and newborn care services. Previous evidence has consistently shown that improved maternal healthcare utilization, including ANC attendance and facility-based care, contributes to better child survival outcomes, particularly in low- and middle-income settings [51]. Likewise, the small inverse association for CS may reflect the potential of timely obstetric intervention to prevent adverse birth outcomes in high-risk pregnancies where vaginal delivery may pose greater danger to the mother or child. In such contexts, medically indicated CS can be lifesaving and may contribute to reductions in perinatal and early childhood mortality, even though unnecessary or elective CS has also been linked to neonatal risks in some settings [52].

The regional relative risk estimates further demonstrated clear spatial heterogeneity in under-five mortality, indicating that mortality risk remains unevenly distributed geographically. This pattern is consistent with recent evidence showing that under-five mortality in sub-Saharan Africa is spatially clustered and strongly shaped by contextual inequalities, including differences in maternal education, access to health services, and place-based socioeconomic disadvantage [51].

The consistency of these findings with those obtained from the other spatial models strengthens confidence in the robustness of the identified regional disparities and highlights the need for geographically targeted and equity-focused child survival interventions.

### 4.1. Conclusion

The Northern regions of Ghana consistently demonstrated elevated under-five mortality rates (U5MR) alongside limited maternal health service coverage. This finding underscores a persistent and pronounced North-South divide in maternal and child health outcomes. In contrast, the Southern and Middle-belt regions exhibited more heterogeneous patterns. Some regions maintained low U5MR despite similarly low levels of service coverage. This may reflect the influence of unmeasured protective factors or possible underreporting. Findings from spatial clustering analyses further confirmed this imbalance, with the north repeatedly emerging as a high-risk area. These clusters were often associated with poor access to healthcare and greater rurality, pointing to underlying structural and geographic barriers to child survival. Importantly, the elevated mortality burden in the northern regions persisted even after accounting for economic and governance factors, signaling deep-rooted spatial inequities in health.

Among the explanatory factors considered, antenatal care (ANC) coverage consistently emerged as an influential determinant of U5MR, though its impact varied in direction and strength across regions. Additionally, economic conditions and reproductive health behaviors such as contraceptive use and abortion showed region-specific associations with child mortality risk. These findings underscore the need for targeted, context-sensitive interventions that bridge both health service delivery gaps and broader social determinants in order to reduce under-five mortality equitably throughout Ghana.

### 4.2. Limitations

This study has some limitations that should be acknowledged. The analysis relied on secondary data aggregated at the regional level, which may conceal important within-region heterogeneity and more localized determinants of under-five mortality. In addition, the cross-sectional nature of the data limits causal interpretation, such that the reported associations should not be interpreted as evidence of direct cause-and-effect relationships. The use of DHS and administrative data may also introduce potential biases related to sampling, recall, and measurement error.

Furthermore, the study’s ecological design introduces the possibility of ecological fallacy, whereby associations observed at the regional level may not necessarily hold at the level of individual children, mothers, or households. As such, the findings are best interpreted as area-level or contextual associations that may reflect broader geographic and structural disparities, rather than individual-level causal effects. Nonetheless, the study offers valuable insight into the spatial distribution of under-five mortality in Ghana and provides a useful basis for subnational health planning and geographically targeted interventions. Future research using individual-level or multilevel data would be beneficial to validate and extend these findings.

An important methodological limitation of this study relates to the use of Gradient Boosting and SHAP analysis at the regional level, where only 16 observations were available. While these approaches can offer useful insight into variable importance, machine learning methods are generally more appropriate for substantially larger datasets. In the present study, the combination of a small sample size and multiple predictors raises the possibility of overfitting, thereby limiting the robustness, reproducibility, and external validity of the model outputs. For this reason, the Gradient Boosting and SHAP findings should be regarded as exploratory and hypothesis-generating, rather than as definitive evidence of predictor importance for under-five mortality. These findings should therefore be interpreted cautiously and ideally confirmed in future studies using larger samples or finer geographic units of analysis. Accordingly, the machine learning findings were interpreted only as supplementary evidence and were not used in isolation to draw substantive conclusions regarding the determinants of under-five mortality.

Nonetheless, the study offers valuable insight into the spatial distribution of under-five mortality in Ghana and provides a useful basis for subnational health planning and geographically targeted interventions. Future research using individual-level or multilevel data would be beneficial to validate and extend these findings.

### 4.3. Recommendation

From a policy standpoint, these findings underscore the urgent need for geographically targeted interventions. Expanding access to quality maternal and child health services in the northern regions must be prioritized, alongside broader investments in rural infrastructure, health workforce distribution, and community-based health initiatives. To advance equitable reductions in child mortality nationwide, national and regional health policies must adopt a place-based approach one that accounts for the geographic, socioeconomic, and cultural contexts shaping health outcomes. Strengthening surveillance, addressing data gaps, and engaging local stakeholders will be essential to designing and implementing effective, region-specific policies. Additionally, regions showing paradoxical clusters (e.g., high service coverage with high mortality) warrant quality-of-care assessments to ensure that services provided are both timely and effective.

## Supporting information

S1 Appendix Bivariate analysis (LISA) of child mortality and use of injectable contraceptives, smoking, use of oral contraceptive pills, percentage of rural populationwith statistically significant spatial autocorrelation. **Appendix A:** Bivariate LISA cluster maps for spatial association between U5MR and predictors.
(TIF)

S2 Appendix Bivariate analysis (LISA) of child mortality and GNI and CPI with statistically significant spatial autocorrelation. **Appendix A**: Bivariate LISA cluster maps for spatial association between U5MR and predictors.
(TIF)

S3 Appendix SHAP waterfall plot: variable contributions to child mortality risk prediction for Ahafo (top-left panel), Bono (top-right panel), Savannah (bottom-left panel), and Volta (bottom-right panel) regions. **Appendix B**: SHAP waterfall plot: variable contributions to child mortality risk prediction.
(TIF)

S4 Appendix SHAP waterfall plot: variable contributions to child mortality risk prediction for Bono East (top-left panel), Central (top-right panel), Eastern (bottom-left panel), and Oti (bottom-right panel) regions. **Appendix B**: SHAP waterfall plot: variable contributions to child mortality risk prediction.
(TIF)

S5 Appendix SHAP waterfall plot: variable contributions to child mortality risk prediction for Upper East (top-left panel), Upper West (top-right panel), Western (bottom-left panel), and Western North (bottom-right panel) Regions. **Appendix B**: SHAP waterfall plot: variable contributions to child mortality risk prediction.
(TIF)
